# Supplementary material for: Patterns of Intron Gain and Loss in Fungi
Source: PLoS Biol. 2004 Nov 30;2(12):e422. doi: 10.1371/journal.pbio.0020422 (PMC532390; doi:10.1371/journal.pbio.0020422)
Supplement: Table S1 — Also available at http://genes.mit.edu/NielsenEtAl/. (4.3 MB ZIP). [file pbio.0020422.st001.zip › NielsenEtAl/html/590.html]

AN1207.1.NCU02965.1.MG05615.1.FG01336.1


```
 CLUSTAL W (1.82) Multiple Sequence Alignments - Introns Inserted


Sequence 1: NCU02965.1	468 aa
Sequence 2: MG05615.1	785 aa
Sequence 3: FG01336.1	721 aa
Sequence 4: AN1207.1	796 aa
Alignment Length: 842 aa
Number Identitical Residues: 263 aa
Alignment Score (without introns) 13574


MG05615.1 	MPPTPGSKRQRKPAP---------------GISREDSDDELGDEDLPWEWIYDDDDLQKR
NCU02965.1	------------------------------------------------------------
FG01336.1 	MARAASAEKAKRQLP---------------QNSHDLSDDELASDEPTWEWVYNSTPTTER
AN1207.1  	MSPAAAADDVDSQTRKRNALKRAEQWMTKGGIIREDSDDELGEEDLPWEWIYDTELDNNE
          	 . :..:.       . .: . :..  :..    . :... .... . .   .     . 

MG05615.1 	QQEDGTEADDGTTPRKRKRAQQSRSGPKIVGARMGNFECRIGDTVLLKAEGGSSEAWVGI
NCU02965.1	------------------------------------------------------------
FG01336.1 	NDEPQSD-------RKRRK----VVGDRIVGARIGQFECRIGDALMLKADG-SNEAWVAL
AN1207.1  	ETGKGVQASGETPKSSRRRSSR-QSQRKIIGARTGPFECRIGDTVLLKSPE-PGKDWAGI
          	.     .:.. :.  .   :.         .:  .  ..  ..:   .:   ...   . 

MG05615.1 	ICEFVED----DGE--KAAHFLWFSTEREIR-NKQKKRNDFLP0NELYVSPSWDVNPLAA
NCU02965.1	-------------------------------------------~----------------
FG01336.1 	VCDFIED----DGEGEKAVNFMWFSSEREIR-NKEKKRTDFYP0NELYLSPSWDINPLAS
AN1207.1  	ITEFLEEEDDEDDEPIKSANIMWFASPDEFMSTRNKRRADALP~NEQYLTTDFNVNPLNS
          	  .  .........  .:      ::  .  :. ..   .  . ..   :.. . ..  :

MG05615.1 	INGKAIVMSQTAFLKKYPNGKVPRS---SKDFGKVFICRRGCNTRTASYTDEFVWEDIYR
NCU02965.1	------------------------------------------------------------
FG01336.1 	VNGKAKIMSHDAFLSKYPQGRIPKN---HPDYGKVFVCRRGCNTRTATYTEEFIWEEIYS
AN1207.1  	INGKAQVMSKDAFFARYPNGAAPKSKAELAEYNKCIVCRRGVSQLQGRYTEEFVWEDVYQ
          	 ...:   :  :     ...  . ..:.  . ..   .  . .   .  :..   ..   

MG05615.1 	GTEADVLSLGERIKTETKATRARRKTKAP~SPDVYEFRDDNDGDEDRTLAKLQRTPKKPR
NCU02965.1	-----------------------------~------------------------------
FG01336.1 	G-EGDLFALMDKIKVETKAKRVRRKIRSP~SP----------ADDTYLPPQVPQTPTKTG
AN1207.1  	--QDQILELVDLIKDGLKAAKKRKQADAD0YVD---------TKDKDAEDVVPTTPRKKQ
          	  . .     .  .   .:     .  :    .          ..         :. .  

MG05615.1 	TGLATAVTPSKGGRTPSKPATPSSHRR~I~VVKKHLEFTPLATRTLSPSHHQNSPFQIAR
NCU02965.1	---------------------------~-~MIKRHLEFTPLATRVLSPMHIHNSPYQLAR
FG01336.1 	RGSVAATPTSR-----RSTATPGSRIK~R2SASKRLEFTPLATRKLSPSQVESSPFQIAR
AN1207.1  	K-VANATPQSRR----QKAMTTPSHKR2I~IVKKPLEFTPLGTRVLSPAHFAS-PYRQAR
          	     : . :       .. :. :        .: ******.** *** :  . *:: **

MG05615.1 	AQLHVASVPTSLPCREAEFSEVYSHLEAAITDGTGSCIYISGTPGTGKTATVREVVASLD
NCU02965.1	TQLHVASVPTSLPCREAEFSLVYSHLEAAITDGSGTCIYISGTPGTGKTATVREVVSHLD
FG01336.1 	SRLHVSSVPTSLPCREGEFSLVYSHLEAAISDGTGNCIYISGTPGTGKTATVREVVSRLE
AN1207.1  	NLLHVSAVPDSLPCRKKEFDTVYSHLSAAIMEGTGTCIYISGTPGTGKTATVREVVAQLN
          	  ***::** *****: **. *****.*** :*:*.********************: *:

MG05615.1 	HAVRNDELDDFIFVEINGMKVSDPHQAYSLLWEALKGQRVSPAQALDLLEREFSNPSPRR
NCU02965.1	AAVRADELDDFIFVEINGMKITDPHQSYSLLWEALKGQRVSPSQALDLLEREFSHPSPRR
FG01336.1 	ESVGSDELDDFIFVEINGMKITDPHQSYTLLWEALKGERASPAQALDLLEREFSNPSPRR
AN1207.1  	AAVLAEEMDDFIFVEINGMKVTDPHQSYSLLWEALKGDRVSPSHALDLLDREFSNPSPRR
          	 :*  :*:************::****:*:********:*.**::*****:****:*****

MG05615.1 	VPCVVLMDELDQLVTKNQGVMYNFFNWPGLRHSRLIVLAVANTMDLPERTLSNKISSRLG
NCU02965.1	VPCVVLMDELDQLVTKNQGVMYNFFNWPGLRHSRLIVLAVANTMDLPERTLSNKISSRLG
FG01336.1 	IPCVVLMDELDQLVTKNQAVMYNFFNWPTLRHSRLIVLAVANTMDLPERTLSNKISSRLG
AN1207.1  	VSCVVLMDELDQLVTKNQSVMYNFFNWPALRHSRLIVLAVANTMDLPERTLSNKISSRLG
          	:.****************.********* *******************************

MG05615.1 	1LTRITFPGYTHEQLMKIIQSRLEGVPGNIVDPDAIQFASRKVAAVSGDARRALDICRRA
NCU02965.1	~LTRITFPGYNHEQLMRIVQSRLEGVPGDIVDADAVQFAARKVAAVSGDARRALDICRRA
FG01336.1 	1LTRITFPGYNHEQLMRIIQSRLEGVPGNIVDPDAIQFASRKVAAVSGDARRALDICRRA
AN1207.1  	~LTRITFPGYKHTDLMEIITTRLASVPGKIVDSDAIQFASRKVAAVSGDARRALDICRRA
          	 *********.* :**.*: :** .***.***.**:***:********************

MG05615.1 	VELAEAEARDM---------DNETPDTPSKRKQKQ--------LAAAEDDAAAKKKRRGA
NCU02965.1	VELAEADAKVV---------AEAEPPTPSKKK-------------GVEDKVVSKKKG-SA
FG01336.1 	VELAEADAP-------------IDPSTPSKRD--------------------PQTQSKGS
AN1207.1  	VEIAEQSNEAAGVEALDADDTESLPPTPSKTAARKERANAKQTLSISPQKSTATPRKQTP
          	**:** .    . .: .:.. .  * ****    .. :.:..: :   ..  .  :   .

MG05615.1 	GRVTIDTIRRAIAEATSSPLQQYLRALPFSSRLLLAALLVRVTRTGLAESTYGDVLEEMQ
NCU02965.1	GRVTIETVRRAINEATSNPLQQYLRGLPFASRLLLTALLLRTQRTGLAESTFGDVLEEMQ
FG01336.1 	GRVTIATIKKAINEATTNPIQQHLRSLPLMSKLVMAALLLRIRRTGLAETTFGETLDEIH
AN1207.1  	GRVTIATIKQAIQEATSTPLQQSLRCLPLSAKLFLAALLARVRRTGITESTFGDVTDEAK
          	***** *:::** ***:.*:** ** **: ::*.::*** *  ***::*:*:*:. :* :

MG05615.1 	RAVKFASGSRQVELLDRRRIDAAGRYVADPAAAASEEGGGWVSSSAKKKKDQVMHRLAGL
NCU02965.1	RMIKLATGG------DSRPLNAL---MATAGVSAADYGDGIMGG-PRSKPDVRQMRPGGL
FG01336.1 	RAS-------------LRPPPAL------PGVAAVLNSG--LKG-TMTSGQRPMVRPGHI
AN1207.1  	RIAD-------------------------AAVAVAGAAGAGIKEFLLAGGTG--TRVQAL
          	*  .                         ...:.   ... :             *   :

MG05615.1 	SLAAVELTGAGIVNLEAH-------------RAERPSKIRLAVGDEEVRLAFRDDPEIKA
NCU02965.1	AIAAVDLTGAGVINLEAH-------------KAERPSKIRLAVGDEEVRLAFRDDPEIKT
FG01336.1 	HTAALELVAAGLINLEAQ-------------RAERSSKLRLSIADDEVKLALRDDGDLKA
AN1207.1  	GFAAMELMNSGVLALEATSGSRGLGGAAIPTRGDRSGKVRLRVAAEDVRAAFRDDVEAKG
          	  **::*  :*:: *** :.: . ..:: .::.:*..*:** :. ::*: *:*** : * 

MG05615.1 	LGLVL--
NCU02965.1	LGVLL--
FG01336.1 	LGIGV--
AN1207.1  	LGLGMEA
          	**: :.:
```
